# Combination of a multiplex pneumonia panel and Gram staining for antimicrobial selection to treat lower respiratory tract infection

**DOI:** 10.1186/s41479-024-00125-z

**Published:** 2024-03-05

**Authors:** Hiroshi Matsuura, Koudai Arimoto, Yoshihito Takahashi, Masafumi Kishimoto

**Affiliations:** Osaka Prefectural Nakakawachi Emergency and Critical Care Center, 3-4-13 Nishiiwata, Higashiosaka, Osaka, 578-0947 Japan

**Keywords:** Drug resistance, Mechanical ventilation, MRSA, Bacterial pneumonia, Antibiotics, Biofire filmarray pneumonia panel

## Abstract

**Aim:**

This study aimed to examine the utility of simultaneously performed the Film Array pneumonia panels (pneumonia panels) and Gram staining with the same specimens and evaluate their effect on antimicrobial selection.

**Methods:**

This prospective study, conducted from April 2022 to January 2023, enrolled adult patients with pneumonia, including those with ventilator-associated pneumonia (VAP). Specimens obtained at the time of sputum culture were tested using Gram staining and the pneumonia panel. The patients’ characteristics and pneumonia panel results were assessed. We also evaluated the selection of antimicrobial agents for drug-resistant bacteria detected by the pneumonia panel.

**Results:**

This study comprised 39 patients: 25 patients (64.1%) underwent intubation, including 7 (17.9%) patients with VAP. Most tests were performed at the time of admission, while some were performed during hospitalization. Good quality sputum was obtained from intubated patients. The pneumonia panel detected drug-resistant bacteria in 12 cases. Six patients required antimicrobial escalation, while the antimicrobial regimen remained unchanged for 2 patients in whom Pseudomonas aeruginosa was detected and had already received meropenem. The attending physician did not change the antimicrobials, considering the results of Gram staining and the patient’s general condition in 4 patients.

**Conclusions:**

The pneumonia panel might be useful for detecting drug-resistant organisms at an early stage. It may be important to take the Gram staining results and the patient’s condition into account with pneumonia panel for appropriate antibiotic prescription.

## Introduction

The identification of the organisms responsible for bacterial pneumonia is critical for the selection of effective antimicrobial agents. Culture tests are used to identify the causative organisms and their susceptibility to antibiotics, but it usually takes several days to a week to obtain results, and even longer for medical institutions that cannot perform these tests at their facilities. Early identification of the causative organism by the pneumonia panel and detection of drug-resistance genes within hours may facilitate early administration of effective antimicrobial agents, thus improving the outcomes of pneumonia treatment. The benefits of pneumonia panels have already been reported [1–7] and a randomized controlled trial comparing pneumonia panels with standard diagnostic protocols is currently underway [8]. Sputum quality, which is one of the most important factors influencing the results of pneumonia panels, can be evaluated using Gram staining. Gram staining of sputum provides rapid results and is useful in selecting antimicrobial agents and significantly reducing the use of broad-spectrum antibiotics in patients with ventilator-associated pneumonia (VAP) [9]. However, it is difficult to detect drug-resistant bacteria using Gram staining alone, which is further encumbered if patients have already been treated with antimicrobial agents and/or transferred to another hospital. The results of Gram staining can determine the quality of sputum and are very useful for diagnosis, including evaluation of the causative organisms and efficacy of current antimicrobial agents. No study has simultaneously performed pneumonia panels and Gram staining of sputum specimens obtained from patients with pneumonia. The same specimen can be used for the pneumonia panel and Gram staining, and we hypothesized that combining the results of both tests would enable better selection of antimicrobial agents (in accordance with the results obtained from each test, including the quality of sputum). Therefore, the purpose of this study was to simultaneously examine pneumonia panels, culture tests, and Gram staining of the same specimen and evaluate their effect on antimicrobial selection.

## Patients and methods

### Study design

This single-center, prospective study was conducted between April 2022 and January 2023. The study enrolled patients who were hospitalized and treated at our center, and underwent a sputum culture test for pneumonia and treatment with antimicrobial agents. Patients who were newly diagnosed with pneumonia during hospitalization and those with VAP were also included. The specimens obtained at the time of sputum culture were tested using Gram staining and the pneumonia panel. This research was conducted in accordance with the Declaration of Helsinki. The Institutional Review Board for Clinical Research of the hospital provided approval for this study (approval no.: 02-0765-A).

### Treatment protocol for pneumonia

Blood culture, sputum culture, and Gram staining of sputum are performed and sulbactam/ampicillin (SBT/ABPC) is the first-line antiobiotic if there is no risk of bacterial resistance, while ceftriaxone (CTRX) is considered if there is renal dysfunction. If atypical pneumonia is suspected, other antimicrobial agents are administered at the discretion of the attending physician. If patients are suspected of having septic shock or bacterial resistance, antimicrobial agents providing coverage against Methicillin‐resistant Staphylococcus aureus (MRSA) or Pseudomonas aeruginosa are selected at the discretion of the attending physician; if antimicrobial agents have already been administered, antimicrobial agents providing coverage against resistant bacteria are administered at the attending physician’s discretion. In this study, antimicrobial agents were selected based on the results of the pneumonia panel in addition to the above-mentioned tests.

### Film array pneumonia panel

**Fig. 1:**
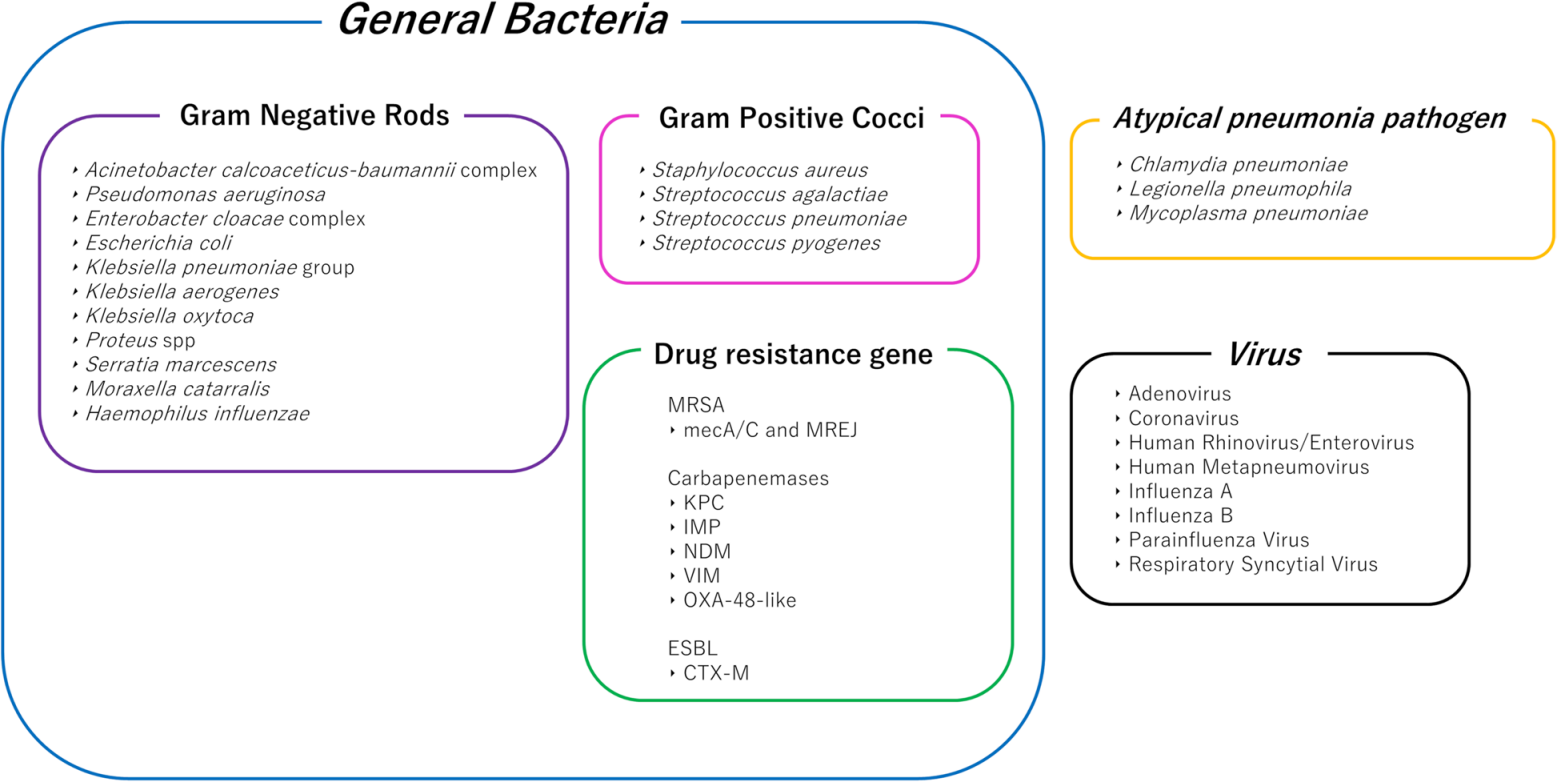
List of detection targets in the pneumonia panel

The Film Array Pneumonia Panel (pneumonia panel) is a cartridge-based multiplex polymerase chain reaction (PCR) assay that includes all steps of molecular diagnostics in an automated manner. In this study, each specimen was analyzed according to the manufacturer’s instructions. The pneumonia panel is compatible with all other Film Array panel platforms [1] such as the Meningitis/Encephalitis [10, 11] and blood culture panels [12, 13]. The results were obtained in a few hours. The 33 items that can be identified by the pneumonia panel are shown in Fig. 1. Gram staining and bacterial culture were also performed using the same specimens.

### Evaluation of the pneumonia panel results

Selection of antimicrobial agents against drug resistance bacteria.

Patients in whom drug-resistant bacteria were detected by the pneumonia panel were divided into three groups.

(i) Patients with antimicrobial agents administered according to protocol
(ii) Patients in whom the pre-test antimicrobial agents were not changed after testing
(iii) Those who received antimicrobial escalation

The attending physician was responsible for the selection of antibiotics based on the assessment of the results of the pneumonia panel, Gram staining, and the patient’s condition.

The patient flow was created based on the choice of antimicrobial agents for drug resistance bacteria using the pneumonia panel.

### Data collection

Patients were followed up until hospital discharge or death. Patient information was collected from the medical records, which included demographic characteristics, laboratory test results, and details of antimicrobial agents before and after sputum investigation.

Clinical data such as the percentage of septic shock, ratio of the partial pressure of arterial oxygen to fractional inspired oxygen (P/F ratio) at specimen collection and the number of patients with VAP were also extracted.

### Statistical analysis

The patient age and other demographic data were presented as the median ± interquartile range (IQR) or counts (percentages). Other variables, such as severity scores, were expressed as the median with the IQR. Statistical analyses were conducted with JMP Pro 16.2 for Windows (SAS Institute Inc., Cary, NC, USA).

## Results

### Patient characteristics and number of bacteria detected

**Fig. 2:**
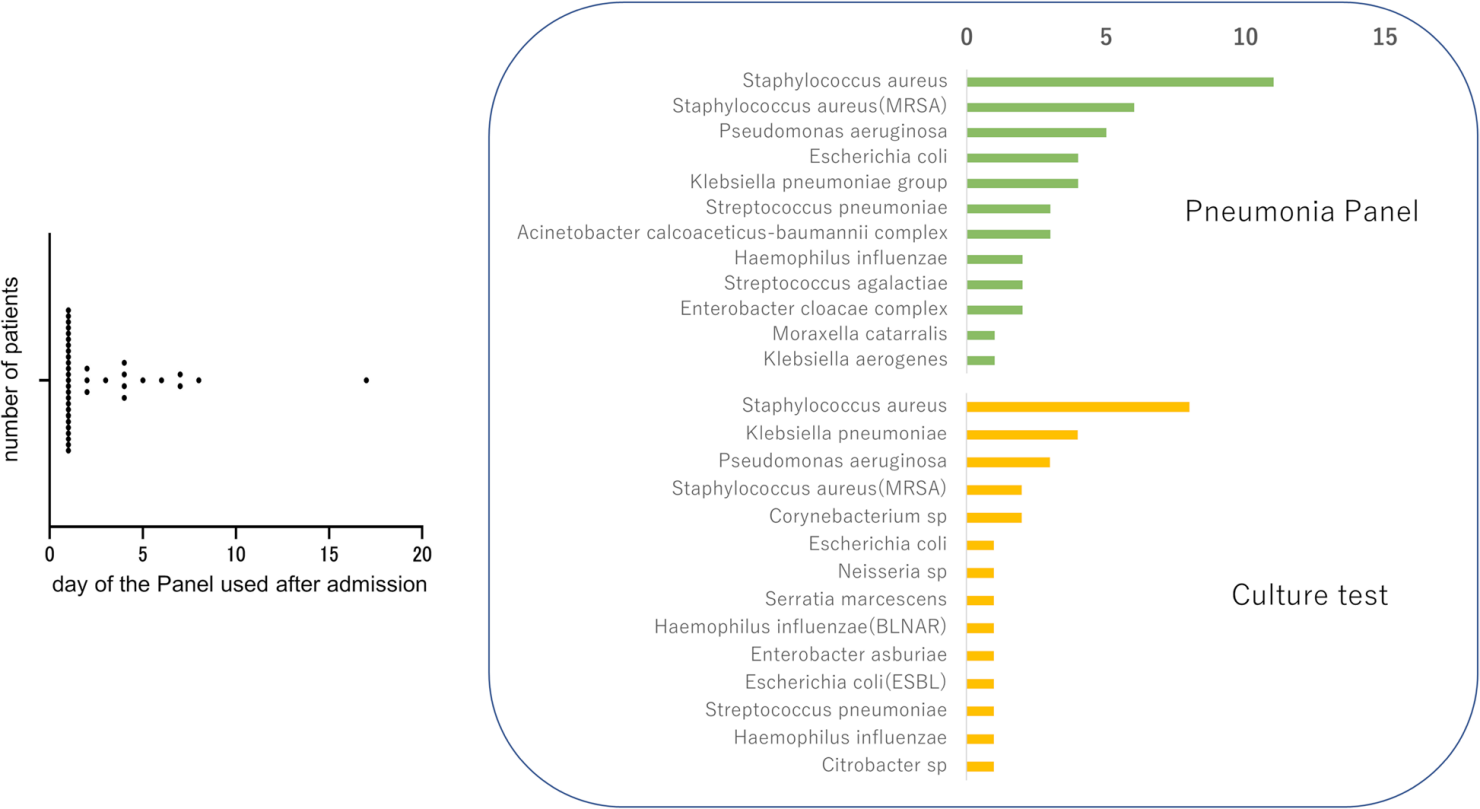
The day of panel testing after admission and the number of bacteria detected by the pneumonia panel and culture test

This study comprised 39 patients. Twenty-five (64.1%) patients were under intubation, including 7 (17.9%) patients with VAP (Table 1). The graph of the number of patients against the days on which the pneumonia panel tests were performed since admission is shown in Fig. 2. Most tests were performed at the time of admission, while some were performed during hospitalization. The total number and bacterial species detected by the pneumonia panel and culture are also shown in Fig. 2.

**Table 1:** Characteristics of the patients used the pneumonia panel

	Total
	*N* = 39
Age, median (IQR)	77 (63–82)
Sex, n (%)	
Male	25 (64.1)
Female	14 (35.9)
BMI (kg/m2)(IQR)	20 (16.8–22.5)
Intubation, n (%)	25 (64.1)
P/F ratio (IQR)	216 (132–304)
Ventirator associated pneumonia, n (%)	7 (17.9)
SOFA score, n (%)	6 (4–7)
HbA1c, median (IQR)	6.1 (5.8–6.8)
Septic Shock, n (%)	14 (35.9)
Antibiotics use before the test	13 (33.3)
Geckler classification 4 or 5	22 (56.4)
Number of bacteria detected by panel	1.2 (± 1.1)
Number of patients detected drug-resistant bacteria	12 (30.1)
Mortality, n (%)	7 (17.9)

### Selection of antimicrobial agents for drug-resistant bacteria by the pneumonia panel

**Fig. 3:**
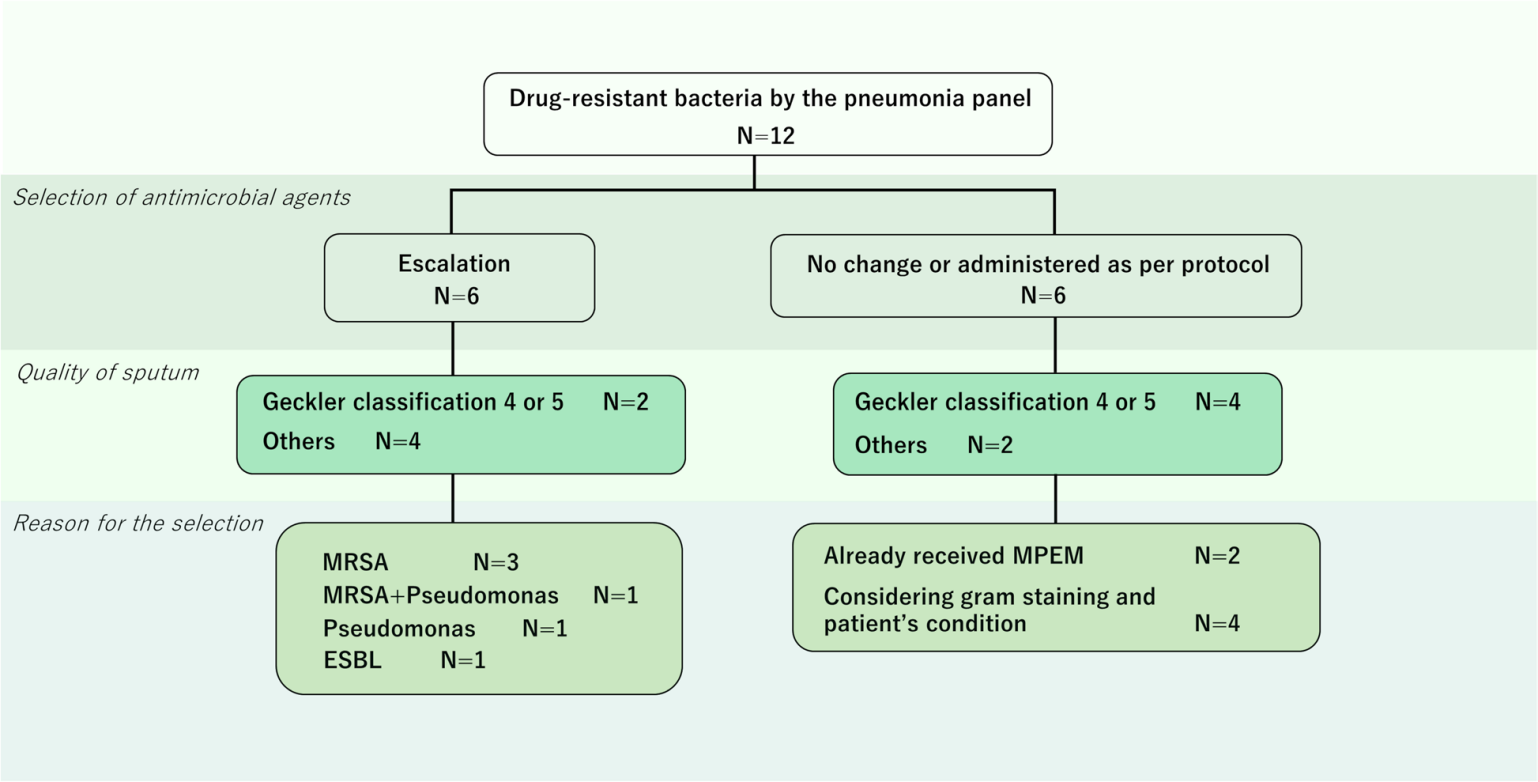
Flow chart detailing the patients’ clinical course: Comparison between the pneumonia panel and culture test, and choice of antibiotics

Drug-resistant bacteria were detected in 12 patients by the pneumonia panel (Table 2): MRSA was detected in 6 patients, Pseudomonas aeruginosa in 5 patients, and extended-spectrum beta-lactamases (ESBL)-producing bacteria in 3 patients. Escalation of antimicrobial therapy was instituted in 6 patients as follows: anti-MRSA drugs for 4 patients with MRSA infection, tazobactam/piperacillin and cefepime for 2 patients with Pseudomonas aeruginosa infection, and meropenem for ESBL-producing bacteria. The antibiotics were not changed in 2 patients who had already received meropenem and in whom Pseudomonas aeruginosa was detected by the panel. The panel detected drug-resistant bacteria in the remaining 4 patients, but the antimicrobials were not changed by the attending physician, considering the results of Gram staining and the patient’s general condition (Fig. 3). One of these 4 patients (patient 4) had pneumonia and urinary tract infection, and the urinary tract was judged to be the focus of infection; thus, antimicrobial agents for the latter were continued. ESBL-producing E. coli was detected by the panel in patient 5, but the number of bacteria detected was low at 10^4^ copies/mL, and Gram staining revealed gram-positive cocci (GPC) 4 + and gram-negative rods (GNR) 1 + ; thus, the GPC was judged to be the focus of infection and antimicrobial agents were administered according to protocol. The panel detected MRSA in patient 6, but Gram staining showed a low bacterial count; thus, antimicrobials were administered as per protocol based on the general condition. ESBL-producing E. coli was detected in patient 10; however, few E. coli were detected at 10^4^ copies/mL, while Gram staining revealed GPC 4 + and GNR 1 + . Thus, the GPC were judged to be the focus and SBT/ABPC was administered as per protocol. Antibiotic escalation was not required in 3 patients with good quality sputum and 1 patient whose sputum quality was designated as “other” (Table 2). On the other hand, the β-lactamase negative, ampicillin resistant (BLNAR) strains of Haemophilus influenza could have been detected at our hospital, and the antimicrobial agent was changed to CTRX in the 2 patients where it was detected.

**Table 2:** Patients characteristics detected drug-resistant bacteria and choice of antibiotics

No.	Pneumonia Panel			Culture test		Gram Stain					Geckler	Septic shock	Antibiotics used before the test	Choice of antibiotics
	Resistance gene	Detected pathogens	c/mL	Detected pathogens		GNC	GPC	GNR	GPR	Fungus				
1		Staphylococcus aureus	>10^7^	Staphylococcus aureus	1+	-	2+	-	-	-	5	-	MEPM	No change (MEPM)
		Pseudomonas aeruginosa	10^4^											
		Streptococcus agalactiae	10^6^											
2		Escherichia coli	>10^7^	Klebsiella pneumoniae	3+	-	4+	4+	4+	4+	4	+	ー	Escalation (LZD)
		Klebsiella pneumoniae group	>10^7^	α-Streptococcus sp	2+									
	mecA/C and MREJ	Staphylococcus aureus	>10^7^	Candida sp	3+									
		Streptococcus agalactiae	>10^7^											
		Streptococcus pneumoniae	>10^7^											
3		Pseudomonas aeruginosa	>10^7^	Pseudomonas aeruginosa	1+	-	4+	1+	1+	-	5	+	ABPC/SBT	Escalation (TAZ/PIPC)
4		Pseudomonas aeruginosa	>10^7^	Pseudomonas aeruginosa	1+	-	1+	2+	-	-	5	-	CMZ	No change (CMZ)
	mecA/C and MREJ	Staphylococcus aureus	10^5^											
5	CTX-M	Escherichia coli	10^4^	Escherichia coli(ESBL)	1+	-	4+	1+	-	-	5	-	CEZ	Protocol (ABPC/SBT)
		Streptococcus pneumoniae	10^6^	Streptococcus pneumoniae	2+									
6	mecA/C and MREJ	Staphylococcus aureus	10^6^	Staphylococcus aureus(MRSA)	1+	-	1+	1+	-	3+	4	+	ー	Protocol (ABPC/SBT)
		Enterobacter cloacae complex	10^5^	Candida sp	1+									
7		Pseudomonas aeruginosa	10^4^	Pseudomonas aeruginosa	1+	-	1+	-	-	1+	-	-	MEPM	No change (MEPM)
				α-Streptococcus sp	1+									
				Candida sp	1+									
8		Klebsiella pneumoniae group	>10^7^	Klebsiella pneumoniae	2+	-	2+	2+	1+	-	3	-	ー	Escalation (VCM+CFPM)
		Pseudomonas aeruginosa	10^5^	Candida sp	1+									
	mecA/C and MREJ	Staphylococcus aureus	>10^7^	Staphylococcus aureus	2+									
9		Moraxella catarralis	10^4^	Corynebacterium sp	2+	-	1+	2+	2+	1+	3	-	ー	Escalation (LZD)
	mecA/C and MREJ	Staphylococcus aureus	10^4^	Candida sp	1+									
10		Staphylococcus aureus	10^4^	Staphylococcus aureus	2+	1+	4+	1+	1+	-	3	+	ー	Protocol (ABPC/SBT)
	CTX-M	Escherichia coli	10^4^	α-Streptococcus sp	3+									
11	CTX-M	Acinetobacter calcoaceticusbaumannii complex	10^6^	Citrobacter sp	3+	-	2+	4+	4+	-	3	-	ABPC/SBT	Escalation (MEPM)
				Corynebacterium sp	3+									
12	mecA/C and MREJ	Staphylococcus aureus	>10^7^	Staphylococcus aureus(MRSA)	3+	-	3+	-	3+	-	3	-	ー	Escalation (LZD)
		Human Metapneumovirus		α-Streptococcus sp	3+									

*c/mL* copies/mL, *BLNAR* β-lactamase negative ampicillin resistance,* ESBL* extended-spectrum β-lactamase, *MRSA* Methicillin-resistant Staphylococcus Aureus *GNC* gram negatibe cocci, *GPC* gram positive cocci, *GNR* gram negative rod, *GPR* gram positive rod

## Discussion

In this study, pneumonia panel tests were performed in combination with culture tests and Gram staining, and the selection of antimicrobial agent(s) was investigated, especially when resistant organisms were detected. The administration of antibiotics might be appropriately determined for each patient based on the collation of the results of Gram staining and the patient’s condition.

The pneumonia panel is a highly sensitive test, whose results are reportedly supported by quantitative PCR testing and sequencing analysis in 871 of 875 samples that were panel positive and culture negative (manufacturer’s instructions). Moreover, not only is the test capable of quickly identifying the causative organism, but can also simultaneously detect drug resistance genes, making it extremely useful in the treatment of acute pneumonia. However, specimen quality is extremely important since it determines the overall quality of the test, and each facility should develop its own rejection rules (manufacturer’s instructions). As shown by the results of this study, the best indication for the pneumonia panel is patients with pneumonia under intubation because good quality sputum can be obtained from these patients. However, it is difficult to make poor sputum quality an absolute contraindication for the panel test. This is because it is difficult to obtain good quality sputum from non-intubated patients, and the quality of sputum in patients with aspiration pneumonia is inherently poor.

**Fig. 4:**
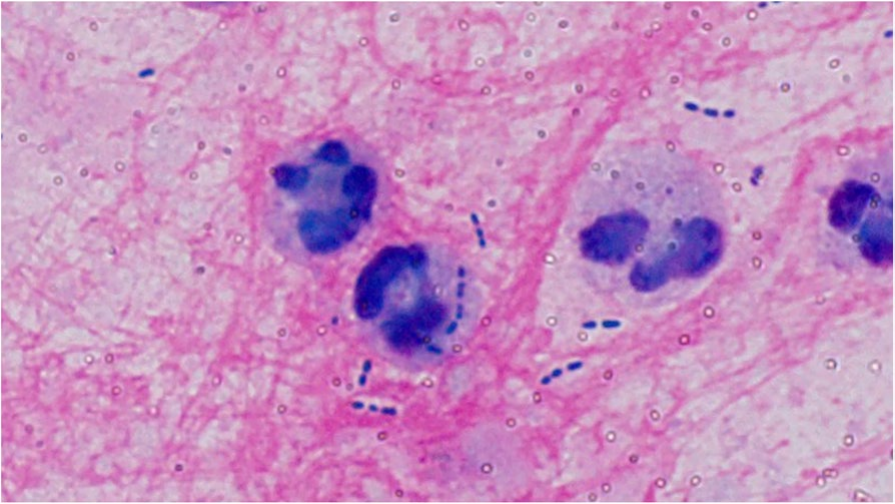
Gram staining showing presence of GPC and phagocytosis by leukocytes

In this study, Gram staining was performed concomitantly with the pneumonia panel. The quality of the sputum was evaluated, and the results of each test could be confirmed for better selection of the antimicrobial agent(s). Three specific patterns are possible. First, when Gram staining of a good quality sputum specimen is indicative of the presence of an inflammatory causative organism, the pneumonia panel can confirm whether the organism is potentially drug resistant, which will aid in the selection of the antimicrobial agent. Second, when a drug resistance gene is detected in the pneumonia panel, a decision is made whether or not to cover the resistant organisms on the basis of the sputum quality, bacterial species, and phagocytosis by leukocytes determined by Gram staining (for example Fig. 4) and the general condition of the patient. Third, if resistant bacteria are detected when the quality of sputum is poor in a patient, we should consider whether a patient have aspiration pneumonia or not. Thus, antibiotic escalation may be selected depending on the patient’s condition. In summary, the pneumonia panel is a highly sensitive test, and not all species detected in the panel are targets for treatment. Confirmation of the species, number of bacteria, and phagocytosis by leukocytes by Gram staining can better define the treatment target, and it is particularly useful in detecting resistant bacteria in the pneumonia panel. Thus, the combined use of the panel and Gram staining is expected to lead to better selection of antimicrobial agents.

Although several studies have investigated pneumonia panels over the past few years [1, 2, 7, 12, 14], no study has examined them in conjunction with Gram staining. Combination with Gram staining may lead to better selection of antimicrobial agents, and we expect to our study to form the foundation of further research in the future.

There are several limitations to this study. First, the sample size was small and the study was conducted at a single institution, making generalization of the findings difficult. Second, we were unable to examine whether aspiration pneumonia was related to the quality of sputum. It is important to enroll a large sample population and conduct a detailed multicenter study in the future.

## Conclusions

We examined the causative organisms and antimicrobial agents in patients with pneumonia using the pneumonia panel and Gram staining. The pneumonia panel might be useful for detecting resistant organisms at an early stage. In addition, it may be important to take Gram staining and the patient’s condition into account with pneumonia panel to ensure appropriate use of antimicrobial agents.

## Data Availability

The datasets used and/or analyzed during the current study are available from the corresponding author on reasonable request.
